# Fetal magnetic resonance imaging diagnosis of pulmonary lymphangiectasia in hypoplastic left heart syndrome: Association with fetal echocardiography and postnatal outcome

**DOI:** 10.1016/j.jocmr.2025.101984

**Published:** 2025-11-04

**Authors:** Greg Leonard, Alexia Egloff, Gema Priego, Tomas Woodgate, Wendy Norman, Milou PM van Poppel, Johannes Steinweg, Thomas Day, Vita Zidere, Owen Miler, Reza Razavi, John M. Simpson, Trisha Vigneswaran, Kuberan Pushparajah, David F.A. Lloyd

**Affiliations:** a School of Biomedical Engineering and Imaging Sciences, King’s College London, London, United Kingdom; b Department of Radiology, Guy’s and St Thomas’ NHS Foundation Trust, London, United Kingdom; c Department of Congenital Heart Disease, Evelina London Children’s Hospital, London, United Kingdom

**Keywords:** Congenital heart disease, MRI, Fetal, Pulmonary lymphangiectasia

## Abstract

**Background:**

Secondary pulmonary lymphangiectasia (PL) is a recognised complication of hypoplastic left heart syndrome (HLHS) with an intact or restrictive atrial septum, associated with poor postnatal outcomes. Fetal MRI has been increasingly used to assess pulmonary abnormalities in HLHS, but the prognostic significance of subtle PL-like changes remains unclear. In this study, we evaluate the relationship between fetal MRI lung findings, echocardiographic markers of pulmonary venous obstruction, and postnatal outcomes.

**Methods:**

A retrospective analysis of all fetuses with HLHS who underwent fetal MRI between July 2019 and December 2022 was performed. MRI images were reviewed for features of PL and categorised as “normal,” “suspicious,” or “diagnostic” of PL. Pulmonary venous Doppler velocity-time integral (VTI) ratios from the most recent fetal echocardiogram were then compared to MRI findings. Postnatal outcomes, including early ventilation, need for intervention, and survival at 28 days and 1 year, were assessed.

**Results:**

Of 20 fetuses with HLHS who underwent MRI, 6/20 (30%) showed features suspicious or diagnostic of PL (5 “suspicious” and 1 “diagnostic”), and 6/20 (30%) showed some evidence of pulmonary venous obstruction (PVO) on echo. While echo markers of PVO were significantly associated with some degree of PL on MRI (p = 0.006), neither PL nor PVO predicted the need for early support/intervention or survival in fetuses who underwent active postnatal management.

**Conclusion:**

Fetuses with HLHS may exhibit a spectrum of lung changes on fetal MRI related to pulmonary venous obstruction. Whilst technical factors may also play a role, a degree of caution is advisable when interpreting more subtle forms of PL in fetal life, particularly in the absence of echocardiographic markers of severe atrial restriction. Larger, multi-centre prospective studies are needed to refine diagnostic criteria for PL in HLHS and better understand its prognostic significance in terms of both early and long-term outcome.

## Graphical Abstract

**Figure:**
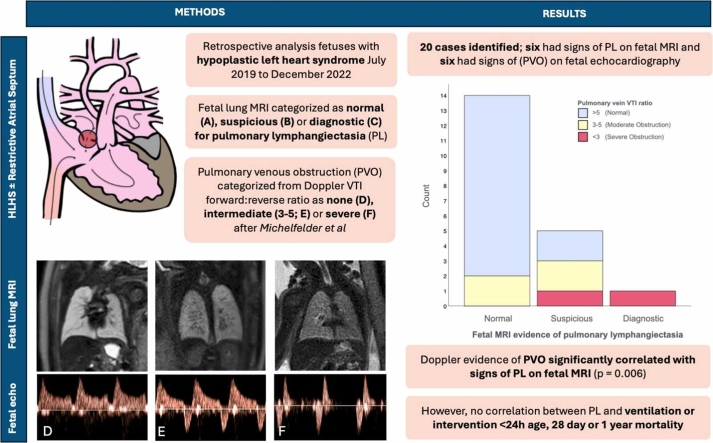

## 1. Introduction

Secondary pulmonary lymphangectasia has been associated with poor outcomes in variants of hypoplastic left heart syndrome (HLHS) with an intact or restrictive atrial septum. Fetal MR evaluation of PL was first reported in 2009 [1], characterised as a T2-hyperintense heterogeneous pattern labelled as “nutmeg lung” [2].

Previous studies have generally described a florid picture of severe PL, often with frank pleural effusions [1], [3] associated with up to 100% mortality/orthotopic heart transplantation prior to the stage 2 procedure [4], [5]. In practice, however, more subtle lung heterogeneity on fetal MRI can be difficult to interpret. In this study, we aimed to evaluate the relationship between prenatal lung MRI findings with fetal echocardiography, early postnatal course, and 1-year mortality.

## 2. Methods

Institutional approval was granted for review of clinical data at Guy’s & St Thomas’ NHS Foundation Trust Hospitals (ref: 10793). Fetal MRI scans performed between July 2019 and December 2022 on fetuses with HLHS (or associated variants where egress of pulmonary venous return was dependent on flow across the foramen ovale) were included. The initial decision to refer for MRI was made at the discretion of the referring fetal cardiologist.

All studies were performed on a 1.5T Philips Ingenia (Philips Healthcare, Best, the Netherlands), including a single shot fast-spin echo (ssFSE) stack acquired in coronal orientation to the fetal thorax (TR 20,000 ms; TE 180 ms, flip angle 90°, voxel size 1.25 × 1.25 mm, slice thickness 2.5 mm; sensitivity encoding factor 2). These sequences had been previously optimised in-house for visualisation of the lung parenchyma, as part of a long-running fetal MRI programme. MRI images were retrospectively reviewed independently by two paediatric radiologists (A.E., G.P.). Reviewers were aware of the prenatal diagnosis but blinded to postnatal outcomes. Corresponding fetal body ssFSE sequences (TE 80 ms) were also reviewed where appropriate (both in their native form and following 3D slice-to-volume registration) to provide additional context and assess overall imaging characteristics. Cases were then categorised into one of three categories for a radiological diagnosis of pulmonary lymphangiectasia: “normal”; “suspicious” (lung fields show abnormal signal heterogeneity; PL cannot be confidently excluded) or “diagnostic” (marked heterogeneity with linear hyperintense regions extending from the hilums +/- pleural effusions). Discrepancies were jointly re-assessed generating a consensus for all cases.

The most recent echocardiogram prior to the MRI was used to obtain the pulmonary vein Doppler velocity-time integral (VTI). The forward: reverse VTI ratio was then calculated as per Michelfelder et al. [6], with a ratio of >5 classified as “unobstructed”, 3–5 as “moderate obstruction”, and <3 as “severe obstruction”. After delivery, postnatal outcome data were collected, including need for early ventilation (<24 h), need for emergent postnatal procedure (<24 h), 28-day and 1-year survival.

Data analysis was performed using SPSS Statistics (Version 29.0.1.0, IBM Corp., Armonk, New York). Univariate logistic regression models were used to describe associations between fetal echocardiographic, MRI findings, and postnatal outcomes where appropriate. Groupwise comparisons of categorical outcomes were assessed using Fisher’s exact test.

## 3. Results

Of 59 cases seen in fetal cardiology over the study period, 20 underwent fetal cardiac MRI: 15 cases with mitral atresia/aortic atresia (MA/AA); 3 cases with mitral stenosis/AA (MS/AA); 1 case with MS/aortic stenosis (MS/AS) and 1 with MS/double outlet right ventricle (MS/DORV). The median age at MRI was 29 years (interquartile range [IQR]: 6 years). The mean gestational age (GA) at MRI was 32.8 weeks (standard deviation [SD]: 1.7 weeks; range 30.1–36.3 weeks); mean GA at delivery was 38.3 weeks (SD: 1.0 weeks; range 36.0–39.4) with mean birthweight 2854 g (SD: 628 g, range 1820g–4040g). One twin pregnancy was included, using imaging from the affected twin only.

### 3.1. Fetal MRI findings

**Fig. 1:**
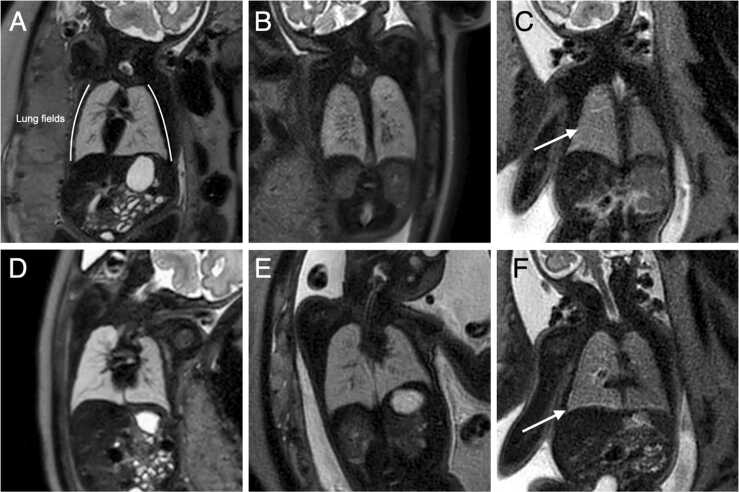
Example T2-weighted fetal MRI images. Panels A and D show images from two cases reported as showing no evidence of PL; panels B and E show images from two cases showing increased generalised lung heterogeneity suspicious of PL; panels C and F show images from a single cases with diagnostic features of PL, including linear hyperintense signal extending from the hilums (C, arrowed) and pleural effusions (F, arrowed). *MRI* magnetic resonance, *PL* pulmonary lymphangiectasis

Following consensus review, 6/20 fetuses (30%) showed MRI evidence of PL. Of these, 5/6 were reported as *“suspicious”* and 1/6 as *“diagnostic” (also associated with pleural effusions)*. Example images are shown in Fig. 1.

### 3.2. Fetal echocardiography

**Fig. 2:**
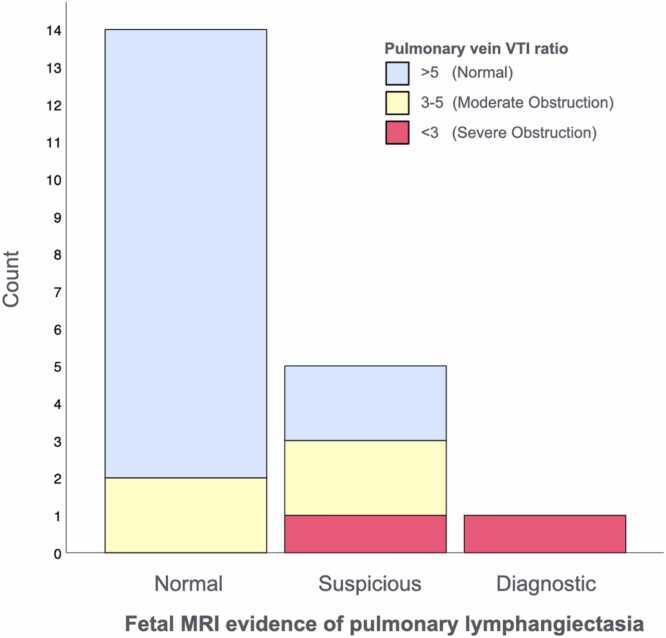
Severity of obstruction of pulmonary vein Doppler VTI compared with severity of pulmonary lymphangiectasia on fetal MRI. *VTI* velocity-time integral, *MRI* magnetic resonance imaging

Of the 14 cases with normal lung appearances on fetal MRI, the majority (12/14; 86%) had an unobstructed PV pattern on echocardiography (VTI ratio >5), with 2/14 (14%) showing signs of moderate obstruction (VTI ratio 3–5) and none with severe obstruction (VTI ratio <3). Of the five cases with “suspicious” findings for PL, 2/5 (40%) had an unobstructed pattern, 2/5 (40%) had a moderate obstructive pattern, and 1/5 (20%) had a severe obstructive pattern. The single case with diagnostic PL findings also had a severely obstructive PV Doppler pattern (Table 1; Fig. 2). There was a significant correlation between increasing PV Doppler severity and increasing PL category (Pearson correlation coefficient 0.59, p = 0.006). Regression analysis gave an odds ratio of 8.9 (p = 0.032) for the development of PL (of any type) for each worsening category of pulmonary venous obstruction.

**Table 1:** Cohort characteristics and postnatal outcome by MRI lung findings.

		Presence of pulmonary lymphangiectasia on fetal MRI			
		*None* *(n = 14)*	*Suspicious* *(n = 5)*	*Diagnostic* *(n = 1)*	*TOTAL* *(n = 20)*
HLHS Subtype	MA/AA	11/14 (78%)	3/5 (60%)^NS^	0	14/20 (70%)
	MS/AA	3/14 (22%)	1/5 (20%)^NS^	0	4/20 (20%)
	MS/AS	0	1 (20%)	0	1/20 (5%)
	MA/DORV	0	0	1/1 (100%)	1/20 (5%)
PV VTI Ratio†	> 5 (Unobstructed)	12/14 (86%)	2/5 (40%)	0	14/20 (70%)
	3–5 (Moderate)	2/14 (14%)	2/5 (40%)	0	4/20 (20%)
	< 3 (Severe)	0	1/5 (20%)	1/1 (100%)	2/20 (10%)
Intention to treat	No	2/14 (14%)	1/5 (20%)	1/1 (100%)	4/14 (20%)
	*Yes*	*12/14 (86%)*	*4/5 (80%)*	*0*	*16/20 (80%)*
Postnatal outcome	Ventilation <24 h	3/12 (25%)	0/4 (0%)^NS^	-	3/16 (19%)
	Procedure <24 h	1/12 (8%)	0/4 (0%)^NS^	-	1/16 (6%)
	28d Survival	11/12 (92%)	3/4 (75%)^NS^	-	14/16 (88%)
	1-year Survival	10/12 (83%)	2/4 (50%)^NS^	-	12/16 (75%)

Groupwise comparisons performed using Fisher’s exact test. *NS* non-significant, *MRI* magnetic resonance imaging, *HLHS* hypoplastic left heart syndrome, *PV* pulmonary venous, *VTI* velocity-time integral, *MA* mitral atresia, *AA* aortic atresia, *MS* mitral stenosis, *AS* aortic stenosis, *DORV* double outlet right ventricle

† Regression analysis demonstrated a significant association between increasing PV VTI severity and the presence of PL on MRI (Pearson r = 0.59, p = 0.006; OR 8.9, p = 0.032) (see also Results)

### 3.3. Relationship to postnatal outcome

After delivery, 4/20 babies (20%) were managed with compassionate care only (two with normal lung appearances, one “suspicious,” and one “diagnostic” of PL). Three were declined for surgery based on postnatal condition; one case with significant co-morbidities (microcephaly and structural brain abnormalities associated with mosaic 45XO/Turner syndrome) had an advanced care directive in place after multi-disciplinary involvement with the patient and their family. Abnormal PV Doppler VTI ratios significantly predicted a non-intervention strategy in this cohort (p = 0.014); however, the presence PL did not (p = 0.174). In the remaining 16 patients, 4/16 (25%) had signs of PL on fetal MRI. Neither the presence of PL or abnormal PV VTI ratio predicted any outcome measure, including need for early ventilation (p = 0.429 and 0.084, respectively), early surgical intervention (p = 0.440 and 0.074), 28 day (p = 0.201 and 0.265), or one-year survival (p = 0.571 and 1.000). Groupwise comparisons are shown in Table 1**.**

Of the two cases with “suspicious” signs of PL that did not survive, one died on the intensive care following cardiac arrest post-Norwood. The second case had a complex course post-Norwood, requiring early bidirectional Glenn procedure at 4 months, followed by catheterisation to occlude multiple aorto-pulmonary collaterals. He was also managed for gastrointestinal failure and died at 10 months of age.

Finally, two cases had an established genetic diagnosis as follows: the case with mosaic 45XO/Turner syndrome and an additional case with Mowat-Wilson syndrome (ZEB1 mutation). Both cases had no evidence of pulmonary venous obstruction on echocardiography and normal lung appearances on MRI. As above, the case with mosaic 45XO and was managed with compassionate care only; the case with Mowat-Wilson syndrome required no early support/intervention and is alive at 1 year.

## 4. Discussion

In this pilot study, we examine the relationship between prenatal imaging and postnatal outcomes in fetuses with HLHS variants where pulmonary venous return is dependent on the foramen ovale. To explore the impact of more subtle changes on fetal lung MRI, we implemented a simple grading system for PL *(“suspicious”* or *“diagnostic”*). We found no significant association between suspected PL on fetal MRI and the need for early respiratory support, surgical intervention, or neonatal or one-year survival. Whilst this may be due at least in part to the small sample size **(**Table 1**)** it is notable that two infants with suspected PL survived to one year. Given previous reports suggesting near-universal early mortality in cases of suspected prenatal PL [1], [2], [4], [5], [7], this suggests that subtle MRI changes alone may not always predict an inevitably fatal prognosis.

Various technical factors (such low signal noise ratio, motion artefact, maternal habitus) have the potential to contribute to a spurious finding of PL where MRI data is limited. However, it is notable that the presence of pulmonary venous obstruction on echocardiography was still a strong predictor of PL on MRI. It has been proposed that chronic pulmonary venous obstruction *in utero* leads to changes in vascularisation and morphology of the pulmonary veins [8] with increased lymphatic drainage and subsequent congestion. In fetal MRI, this is visible as linear and tubular T2-hyperintense structures running from the hila to the pleural surface [2], with severe cases showing pleural effusions or hydrops [3]. However, the relationship between PV obstruction and PL is not always predictable and potentially influenced by other factors that remain unclear [2], [4], [9].

Our findings align with previous observations [10], suggesting that caution is warranted when interpreting subtle lung changes in HLHS, particularly in the absence of echocardiographic evidence of pulmonary venous obstruction. This raises important implications for parental counselling and early postnatal management where PL is suspected, as well as considerations around patient selection for prenatal septostomy, which has been described in the presence of severe PL [1], but for which standardised criteria have not yet been established.

## 5. Limitations

The sample size in this pilot study is relatively small, limiting statistical power. As MRI was performed at the discretion of the referring fetal cardiologist, a degree of selection bias cannot be excluded. Interpreting radiologists were not fully blinded to prenatal imaging findings, which may have introduced biases in image interpretation; intra-observer variability was not assessed. Future collaborative studies across multiple centers, incorporating multimodal imaging, postnatal histology, and advanced methods such as T2 signal analysis, will be important to further clarify the role of fetal MRI in the detection of PL.

## 6. Conclusion

Fetuses with HLHS may exhibit a spectrum of lung changes on fetal MRI related to pulmonary venous obstruction. Whilst technical factors may also play a role, a degree of caution is advisable when interpreting more subtle forms of PL in fetal life, particularly in the absence of echocardiographic markers of severe atrial restriction. Larger, multi-center prospective studies are needed to refine diagnostic criteria for PL in HLHS and better understand its prognostic significance.

## Author contributions

**Greg Leonard:** Writing – review & editing, Writing – original draft, Visualization, Validation, Methodology, Investigation, Formal analysis, Data curation. **Alexia Egloff:** Writing – review & editing, Visualization, Validation, Methodology, Investigation, Data curation. **Gema Priego:** Writing – review & editing, Visualization, Validation, Methodology, Investigation, Data curation. **Tomas Woodgate:** Writing – review & editing, Visualization, Validation, Methodology, Investigation, Formal analysis, Data curation. **Wendy Norman:** Writing – review & editing, Supervision, Software, Resources, Project administration, Methodology, Investigation, Funding acquisition. **Milou PM:** van Poppel Writing – review & editing, Methodology, Investigation, Data curation. **Johannes Steinweg:** Writing – review & editing, Formal analysis, Data curation. **Thomas Day:** Writing – review & editing, Supervision, Resources, Methodology, Investigation, Data curation. **Vita Zidere:** Writing – review & editing, Supervision. **Owen Miler:** Writing – review & editing, Supervision. **Reza Razavi:** Writing – review & editing, Supervision. **John M. Simpson:** Writing – review & editing, Supervision. **Trisha Vigneswaran:** Writing – review & editing, Supervision, Formal analysis, Data curation. **Kuberan Pushparajah:** Writing – review & editing, Supervision, Funding acquisition, Formal analysis, Conceptualization. **David F.A. Lloyd:** Writing – review & editing, Writing – original draft, Visualization, Validation, Supervision, Resources, Project administration, Methodology, Investigation, Funding acquisition, Formal analysis, Data curation, Conceptualization.

## Declaration of Competing Interest

The authors declare that they have no known competing financial interests or personal relationships that could have appeared to influence the work reported in this paper.
